# An Insight Into the Physicochemical Properties of Gold Nanoparticles in Relation to Their Clinical and Diagnostic Applications

**DOI:** 10.7759/cureus.37803

**Published:** 2023-04-18

**Authors:** Houriah Y Nukaly, Shakeel A Ansari

**Affiliations:** 1 Medicine and Surgery, Batterjee Medical College, Jeddah, SAU; 2 Medicine, Batterjee Medical College, Jeddah, SAU

**Keywords:** applications, diagnostic, clinical, properties, gold nanoparticles

## Abstract

The ease of formulation and surface modification of gold nanoparticles (AuNPs) by ligands, greater biocompatibility, non-cytotoxicity, and excellent optical properties are the characteristics that necessitate their application in clinical and genomic research. Not only that, but the extensive synthetic chemistry of AuNPs also offers precise control over physicochemical and optical properties owing to the inert, biocompatible, and non-toxic nature of the inner gold core. Another important property of AuNPs involves their incorporation into larger structures, including liposomes or polymeric materials, thereby increasing their capability of drug delivery in concurrent therapy and imaging labels for enhanced diagnostic applications. AuNPs are endowed with physical properties that suggest their use as adjuvants for radiotherapy and bio-imaging and in computed tomography (CT) scans, diagnostic systems, and therapy. Thus, these features strongly endorse the AuNPs in thrust areas of biomedical fields. The diverse properties of gold nanoparticles (AuNPs) have made them promising candidates in biomedical fields, including in the development of theranostics, which encompasses using these gold nanoparticles for both diagnosis and therapy simultaneously. To appreciate these and related applications, a need arises to review the basic principles and multifunctional attributes of AuNPs in relation to their advances in imaging, therapy, and diagnostics.

## Introduction and background

Nanotechnology entails the study of the molecular and submolecular structural characteristics of nanostructures. It has been used extensively in bionanotechnology based on its electrical, optical, and magnetic characteristics [1,2]. It refers to the development and utilization of materials that are produced at the nanoscale, usually up to 10-1,000 nm in size. The unique properties and multiple surface functionalities make gold nanoparticles (AuNPs) widely used in biotechnology. The multifunctionality of AuNPs makes them a useful material to be assembled with proteins, oligonucleotides, and antibodies [3]. Besides, the development of innovative biomaterials for the study of biological systems has also made AuNP bioconjugates an increasingly viable alternative. Due to the resilience of AuNPs, numerous valuable materials have been provided for a variety of biomedical applications [2,3]. Additionally, AuNPs exhibit the property of having a high surface area, which serves as valuable platforms for therapeutic agents such as drugs and targeting agents. The binding event between the analytes and the AuNPs in diagnostics might impact the physicochemical features of the AuNPs, such as surface plasmon resonance (SPR), conductivity, and redox behavior, yielding detectable signals [3]. Not only that, but gold nanoparticles also serve as a candidate for the delivery of small drug molecules to large biomolecules such as DNA, RNA, and proteins. Some drug molecules can be directly conjugated with gold nanoparticles via physical absorption and ionic or covalent bonding without any alteration of the AuNP monolayer [4]. On the other hand, the delivery of large biomolecules necessitates the functionalization of AuNPs such as PEGylation, peptide and amino acid conjugation, or oligonucleotide functionalization [4]. In this review, we present an overview of the various properties and clinical and diagnostic applications of AuNPs and highlight a few of their recent applications in bionanotechnology.

## Review

Properties and applications of gold nanoparticles (AuNPs)

Tunable Optical Properties

**Figure 1:**
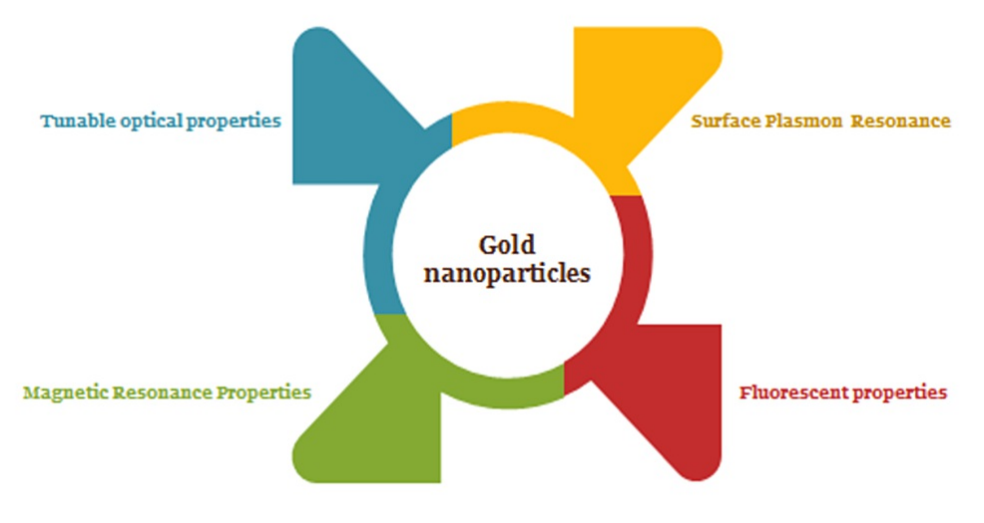
Properties of AuNPs AuNPs: gold nanoparticles

Gold nanoparticles exhibit salient characteristics as illustrated in Figure 1. The optical features of AuNPs are determined by their size and composition. Importantly, the scattering and absorbance characteristics of AuNPs vary with their sizes [1,2]. Studies suggested that AuNPs less than 20 nm exhibited surface plasmon resonance (SPR) with negligible scattering characteristics [3,4]. In contrast, large AuNPs between 20 and 80 nm display increased scattering characteristics of these materials [5-7]. Many studies demonstrated the colloidal nature of spherical AuNPs, which appeared red with surface plasmon resonance (SPR) band observed at 520 nm, which depends on AuNP interparticle distance, surrounding media refractive index, shape, and size [8-10]. The large AuNPs relatively are characterized to show a high-scattering effect [11-13]. Alternatively, the greater absorption characteristics of AuNPs attract their use for colorimetric analyte detection, which is required in biological analysis on the basis of changes in the refractive index of the AuNP environment [11,14].

**Figure 2:**
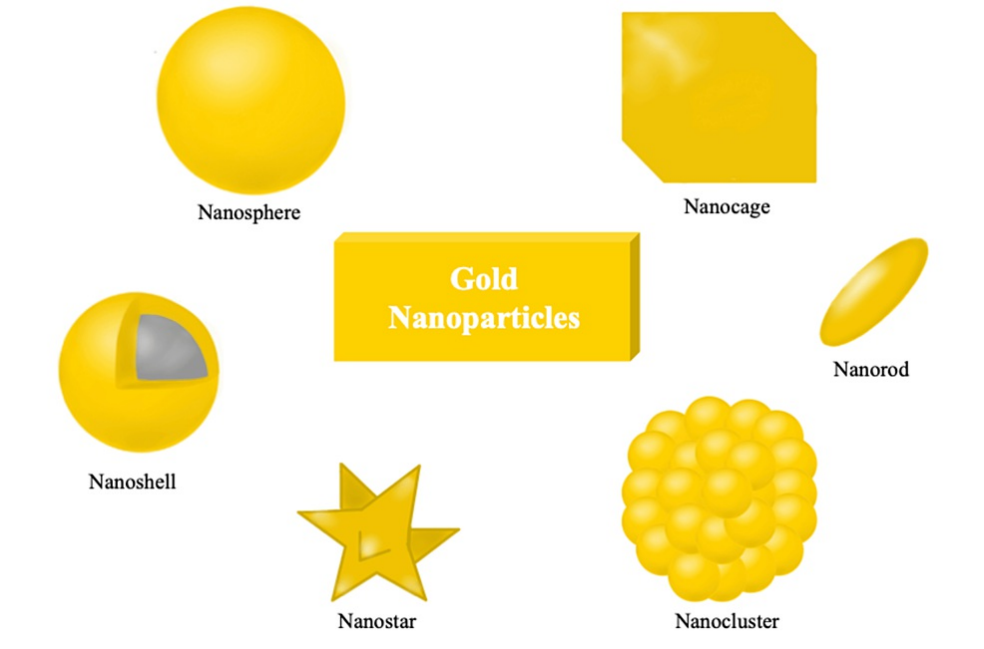
Different morphologies of AuNPs AuNPs: gold nanoparticles

In comparison, the novel concept of making an alloy of Ag-AuNPs imparts the required optical properties for improving the detection of biological interactions based on coupled plasma mass spectrometry (CPMS) and localized surface plasmon resonance (LSPR) [15-17]. The sensitivity of plasmon-based bioassays improves as a result of LSPR, which involves the detection of a single molecule for diagnostic purposes [18]. In fact, Nishimura et al. [18] went a step further and noted that ionophores are located on the sensor in the form of receptors that allowed the specific detection and quantitative analysis of ionic species in biological samples. In this regard, imprinted polymers have been reported as useful for replacing antibodies for the specific and quantitative analysis of small molecules [19]. Figure 2 depicts the various morphological shapes and arrangements of AuNPs that exhibit considerable potential in numerous clinical applications, rendering them a high-potential choice for various medical endeavors. The detection of biomarkers in bio-fluids is promising in point-of-care applications due to its low invasiveness and high adaptability, while the detection of biomarkers in tissues serves as the gold standard for precision diagnosis in pathological examination [20]. The translation of AuNP-based optical diagnostics into clinics requires the modification of existing methods such as enzyme-linked immunosorbent assay (ELISA). Moreover, they can find applications in microflow-controlled chips, which can improve the ability to identify, isolate, and detect targets.

Surface Plasmon Resonance

This phenomenon occurs at the surface of gold (Au) after the incidence of a beam of light at a particular angle and distance, thereby resulting in a gradual reduction in reflected light intensity. By analyzing the refractive index of the surrounding medium on the gold (Au) surface, Englebienne et al. [20] utilized this property to measure the SPR sensitivity, followed by measuring the molecules’ scattering and absorption on the gold surface along their targeted specific ligands. The principal application of this property helped in developing biosensing SPR instruments, which proved useful in determining affinity parameters for biomolecular interactions, especially in diagnostics and therapeutic efficacy. The technology holds promise for detecting small molecules, determining the real-time kinetics of ligand-receptor interactions, and screening lead compound identification in developing pharmaceutical drugs [21]. Many studies surfaced one after another, exploiting these properties while studying DNA hybridization [22,23]. Further studies on enzyme-substrate interactions [24-26], antibody characterization [27,28], antigen-antibody interaction [29,30], and characterization of antibody orientations [31,32] are of note. Many more studies on varied fields such as epitope mapping [33,34], protein conformational studies [35], and label-free immunoassays [36,37] are in extended use. The strong dependence of the SPR effect employing AuNPs finds its way quite successful in bioassay applications, colorimetric sensors, gene therapy, photothermal therapy, and bio-imaging [38,39]. Owing to the remarkable color change from red to purple by AuNPs that is subjected to the change in refractive index, antibodies can easily be attached to AuNPs. Moreover, the analytes bind to the antibodies specifically, which results in a change in color in proportion to analyte concentration [40]. Despite these advances, one of the primary limitations of SPR-based biosensors is that anything that alters the refractive index at the sensing surface will interfere with the analysis, including non-homogenous (complex) sample matrices and nonspecific binding interactions. Hence, research is underway to cope with these issues [41].

Magnetic Resonance Properties

This property paved the way for better molecular imaging, which helps greatly in measuring biological processes at the molecular and cellular levels, therapy, and biological imaging. The utility of AuNPs as template agents provides better magnetic resonance imaging (MRI) contrast agents, owing to their high sensitivity [39,40], and showed improved results in clinical diagnosis [41,42]. All these observations are aimed to quantify molecular changes that are linked to the development and onset of pathological conditions to provide input for early prognosis and diagnosis of cancer. Imaging agents with high density, relaxivity, and ability to target the receptors specifically are required for the imaging of cellular and subcellular structures. Researchers have earlier synthesized the core-shell structured iron-gold nanoparticles (Fe-Au-NPs) through a reverse micelle approach, aimed to analyze their efficacy as magnetic resonance (MR) contrast agents [43]. These AuNPs exhibited superior magnetism and high relaxivity. Further reinforcement comes from a study by Alric et al. [44] when AuNPs were synthesized with high relaxivity for imparting improved contrasting agents for MRI. Moreover, Au cores were encapsulated in a multilayered gadolinium (Gd) organic shell bonded by disulfide bonds, which resulted in the enhancement of contrast while the strong X-ray absorption was provided by Au cores. These AuNPs were revealed to have a dual-modal imaging ability and can freely circulate in the blood vessels without causing an undesirable accumulation in the liver, lungs, and spleen. Also, PEG-coated iron oxide gold nanoparticles (PEG-AuIONs) were developed to show high specificity to solid tumors by accumulating within the mass of the tumor and nonspecifically accumulating in the liver and spleen [44,45].

These studies demonstrate the application of AuNPs as effective MRI contrast agents for the diagnosis of malignant tumors, such as lung and pancreatic cancer. AuNPs were also coated by gadolinium chelate (Gd-Au) as a potential bimodal contrast agent for computed tomography (CT) and MRI with increased efficiency [46,47]. Lack of precise control on monolayer-protected cluster (MPC) stoichiometry and charge state affects the magnetic property of AuNPs. Hence, efforts were raised earlier, which could pave the way to enable controlled magnetism-related applications of gold MPCs, especially those based on the use of molecular MPCs [48,49].

Fluorescence Properties

Biological tests such as fluorescence-based assays and detection techniques are very sensitive in clinical diagnosis. It is because AuNPs, in the presence of strong light illumination, express an excellent anti-photobleaching behavior. As a result, AuNPs, under a high excitation energy state, show strong native fluorescence. The fluorescence of AuNPs inside the cells or on cell membranes can be collected for cell imaging when the cells stained with AuNPs are illuminated with strong light [46]. To monitor intracellular reactive oxygen species (ROS) in viable cells using NP surface energy transfer, Lee et al. [47] examined Au nanoprobes immobilized with fluorescein-hyaluronic acid (HA) conjugates. Also, dopamine was used to robustly prevent the immobilization of HA onto the AuNP surface to secure intracellular stability against glutathione. The advantage of this system is it allows specific and rapid detection of intracellular ROS by releasing strong fluorescence-recovery signals. These results strongly imply that the fluorescence of Au nanoprobes can be used for antioxidant screening and intracellular ROS detection as a new class of ROS imaging probes. In fact, AuNPs are rather useful as fluorescent markers for optical imaging and sensing in analytical genomics and proteomics according to Coto-García et al. [48]. The method strongly emphasizes the different strategies employing AuNPs for bio-imaging and quantitative bioanalysis. A modified technique based on fluorescence, exactly called fluorescence resonance energy transfer (FRET), has shown renewed promise. It is a distance-dependent spectroscopic technique by which the donor electrons’ excitation energy is transferred to the acceptor through an induced dipole-dipole interaction [49]. AuNP based on FRET assay has monitored DNA cleavage and DNA hybridization (DDH) [50]. Even large molecules are also useful for drug screening and protease activity in vivo, such as proteins stabilized by fluorescent imaging probes [51]. You et al. [52] described the use of a fluorescent polymer to decipher the response produced by proteins at nanomolar concentrations via a variety of AuNP-protein affinities. Besides, AuNPs are used as fluorescence quenchers aimed to detect the protein cardiac troponin by its simultaneous interaction with two distinct antibodies, one coupled to AuNPs and the other labeled with fluorescent dyes [53].

Mirkin et al. [54] successfully used the oligonucleotide functionalized AuNPs’ distance-dependent optical properties in colorimetry for DNA detection. Extensive investigation into these nanostructured probes’ characteristics revealed that, in DNA and RNA assays, they display rapid melting transitions when hybridized to complementary DNA. Besides, the catalytic properties of these novel nanoparticles make them useful as signal transducers or amplifiers [55,56]. Since these observations, nanoparticle-based DNA conjugates are frequently used to label DNA (DNA nanoprobes), where a particular nanoparticle tag permits the identification of target molecules [57]. Special thrust was on noble metal nanoparticles of gold, silver, and platinum [58,59]. The AuNPs being the focus of discussion here will be discussed in detail [60]. AuNPs with a size range of 3-100 nm are chosen for better stability and liability and can be tailored easily by chemical modifications [61,62]. Normally charged, these nanoparticles are quite sensitive to dielectric solution changes [63,64].

For citrate-stabilized AuNPs, the addition of NaCl shields the surface charge, resulting in a reduction in the interparticle distance and ultimate particle aggregation [65,66]. Another variant responsible for the intense colors of AuNPs is the SPR [67]. Hence, monodisperse AuNPs in solution appear red, suggesting a quite narrow surface plasmon absorption band, whereas aggregated AuNPs in solution appear blue-purple, showing a distinctive red shift in the SPR to higher wavelengths [57,67]. As a linking molecule, DNA or protein is used to aggregate the AuNPs, allowing biodetection assays to benefit from the optical properties of dispersed gold particles as compared with aggregated gold particles [68,69]. The sensitivity of SPR-based biomolecule sensing methods was improved by AuNPs’ capacity to amplify changes in the SPR of a noble metal surface film when the two were brought in proximity after binding an analyte. Additionally, detection assay sensitivity is improved due to the potential for silver staining of DNA and protein AuNP conjugates and the catalytic reduction of silver ions by AuNPs. Due to their electrical conductivity properties, several chip-based tests have been developed based on electrical read-out systems. Thus, these systems have been used for DNA sequence characterization and single-nucleotide polymorphisms (SNPs) [70].

Mechanistic studies on nanoPCR by Lou and Zhang [71] have added another dimension and diversified studies on AuNPs. These studies highlighted the application of AuNPs in genetic analysis. The surface interaction of PCR components (which includes Taq polymerase, primers, and other products) with AuNPs is proposed to control nanoPCR. Evidently, three typical AuNP effects can explain the nanoPCR mechanism. A simple colorimetric assay and dynamic light scattering measurements directly show that (1) AuNPs adsorb polymerase and regulate the number of active polymerases in PCR, (2) the adsorption of primers by AuNPs reduces the melting temperatures (Tm) of duplexes created with precisely matched and mismatched primers while increasing the Tm difference between them, and (3) AuNPs adsorb PCR products and facilitate their dissociation during denaturation. Hence, all these findings can enhance the PCR of the hepatitis B virus (HBV) gene and the amelogenin genes for genetic testing [54,70,71].

Accounts so far clearly show that AuNPs have a great future in diagnostics. The best-characterized example of AuNPs functionalized with single-stranded DNA (ssDNA) is the exhibition of color change upon aggregation [52]. It can be tailored with various ligands to yield highly selective nanoprobes for diagnosis [52,72]. Even more, when coupled with metal deposition in electrochemical-based methods, it enhances the signal by a notch [8,73,74]. Table 1 provides a summary of AuNP properties [8,73,75-79]. Fluorescent gold nanoparticles were formulated recently in suspension as an efficient theranostic agent for highly radioresistant cancer cells [80]. This suspension was stable in the cellular environment, and the attached fluorophore allowed for a simple location of the nanoparticle. The nanoparticle behaves as expected as a radio enhancer at orthovoltage energies. However, future work requires investigation of the pharmacokinetics and tumor-targeted imaging power of the suspension in live animals to assess the efficacy, sensitivity, and safety of this theranostics tool. One of the groups demonstrated the functionalization of AuNPs with polyaminocarboxylate with near-infrared organic fluorophores (aminated Cy-5) for investigating the utility between subcellular localization and in vivo biodistribution [81]. The developed formulation exhibited improvement in X-ray performances, which could serve as key findings for designing highly efficient nanotheranostic agents [75].

**Table 1:** Properties of AuNPs AuNPs: gold nanoparticles, SPR: surface plasmon resonance, MRP: magnetic resonance property, IMA: ischemia-modified albumin, PTT: photothermal therapy, PDT: photodynamic therapy

SPR property		MRP		Fluorescence properties	
Application	Reference	Application	Reference	Application	Reference
Imaging and phototherapy	[73]	Magnetic cell sorting	[75]	Cancer screening and imaging	[75]
Detection of IMA	[8]	Intracellular tracking	[76]	Fluorescent imaging as DNA biosensing	[79]
PTT	[75]	Drug delivery	[77]	Treatment of cancer	[77]
PDT		Gene therapy	[78]	In vivo therapy of tumors under the skin and deeply seated within tissue	[79]
Colorimetric assays in tumor treatment and diagnosis		Kills cancer cells	[78]	Spectroscopic detection	

## Conclusions

Due to the abovementioned unique features, AuNPs serve as excellent candidates for molecular sensitive detection, effective contrasting agents for molecular imaging, carriers for targeted drug or gene delivery, and therapeutic reagents for specific photothermal therapy. The intrinsic properties of the gold core and the ability to tailor the functionality of their surface are the main characteristic features that make their application ideal in biological systems. However, extensive research requires optimization of the designing of AuNPs as multifaceted vectors for targeting cancer. Further studies are required for understanding the molecular interaction of AuNPs with their target cells (normal as well as malignant) for revealing the mechanism of cancer detection and diagnosis. Future research should prioritize coping up with the chemoresistance and heterogeneity of cancer cells. One such strategy to overcome tumor heterogeneity is by tagging nanoparticles with stromal antagonists. Further investigation is warranted to reveal novel molecular targets that are only expressed in the tumor microenvironment to aid the targeting of nanoparticle-based therapy. Cancer stem cells or cancer-initiating cells can also serve as important candidates for drug targeting. Although AuNPs are inherently non-toxic, it is important to discern the toxicity of the nanoparticle core and that of its capping ligands. The change in pharmacokinetics, biodistribution, and eventual side effects of such conjugated ligands should be eventually considered before suggesting their clinical applicability. Finally, packaging technology needs to be optimized to overcome the obstacles of immunogenicity and tumor penetration.

## Appendices

Abbreviations

AuNPs: gold nanoparticles, SPR: surface plasmon resonance, CPMS: coupled plasma mass spectrometry, LSPR: localized surface plasmon resonance, FRET: fluorescence resonance energy transfer
